# Single-stranded DNA oligonucleotides containing CpG motifs are non-stimulatory *in vitro* but offer protection *in vivo* against *Burkholderia pseudomallei*

**DOI:** 10.3389/fcimb.2024.1458435

**Published:** 2024-10-18

**Authors:** Andrew Scott, Benjamin Farrar, Tom Young, Joann Prior, Chad Stratilo, Leonie Unterholzner, Riccardo D’Elia

**Affiliations:** ^1^ Microbiology, Defence Science and Technology Laboratory, Salisbury, United Kingdom; ^2^ Division of Biomedical and Life Sciences, Faculty of Health and Medicine, Lancaster University, Lancaster, United Kingdom; ^3^ Bio Threat Defence Section, Defence Research and Development Canada, Ralston, AB, Canada; ^4^ Strathclyde Institute of Pharmacy & Biomedical Sciences, University of Strathclyde, Glasgow, United Kingdom

**Keywords:** melioidosis, DNAzyme, CpG, anti-inflammatory, *Burkholderia pseudomallei*

## Abstract

Therapies that modulate and appropriately direct the immune response are promising candidates for the treatment of infectious diseases. One such candidate therapeutic is DZ13, a short, synthetic, single-stranded DNA molecule. This molecule has enzymatic activity and can modulate the immune response by binding to and degrading the mRNA encoding a key immuno-regulatory molecule. Originally developed and entering clinical trials as an anti-cancer agent, DZ13 has also been evaluated as a treatment for viral infections, and has been shown to provide protection against infection with influenza virus in a mouse model of infection. In this work, we evaluated whether the immuno-modulatory properties of DZ13 could provide protection against the potential biothreat pathogen *Burkholderia pseudomallei* which causes the neglected tropical disease melioidosis. Treatment of mice infected with *B. pseudomallei* demonstrated that DZ13 did indeed provide excellent protection after only two post-exposure treatments. However, our data indicated that the enzymatic activity contained in DZ13 was not required for protection, with control oligonucleotide treatments lacking activity against the target mRNA equally as protective against *B. pseudomallei*. We have designed new sequences to study the mechanism of protection further. These novel sequences offer enhanced protection against infection, but are not directly anti-microbial and do not appear to be stimulating the immune system via TLR9 or other key innate immune sensors, despite containing CpG motifs. The molecular mechanism of these novel sequences remains to be elucidated, but the data highlights that these oligonucleotide-sensing pathways are attractive and relevant targets to modulate during bacterial and viral infections.

## 1 Introduction

*Burkholderia pseudomallei* is a Gram negative saprophytic bacterium normally found in soils and surface groundwater (Limmathurotsakul et al., 2013) in tropical and sub-tropical regions (Limmathurotsakul et al., 2016). It is an opportunistic pathogen that can cause disease in a wide range of domestic and wild animal species (Sprague and Neubauer, 2004; Kasantikul et al., 2016), and is the causative agent of the disease melioidosis in humans (see (Wiersinga et al., 2018; Meumann et al., 2024) for recent reviews about *B. pseudomallei* and melioidosis). Disease presentation is highly varied, ranging from localised manifestations at the site of infection to systemic syndromes such as pneumonia, bacteraemia and septic shock. Mortality rates for patients with melioidosis are 10 to 40% (Cheng and Currie, 2005). Although most cases of melioidosis are recorded in Southeast Asia and northern Australia, it is thought that disease is highly underreported and there are estimated to be nearly 100,000 deaths per annum globally (Limmathurotsakul et al., 2016). Early diagnosis and implementation of appropriate treatment are crucial to successfully treating melioidosis; mortality rates are considerably higher in regions where diagnostic and treatment facilities are limited (Currie, 2015). *B. pseudomallei*, and its close relation *B. mallei*, is considered to be a potential agent of bioterrorism or biowarfare, and new treatments are urgently being sought to improve outcomes for naturally acquired infections and to guard against the threat of a deliberate release (Duplantier et al., 2020).

*B. pseudomallei* has intrinsic resistance to many first and second line antibiotics (Thibault et al., 2004), and treatment can be challenging even where it is sensitive. As a case in point, the current standard treatment involves an intensive phase of ten to fourteen days of intravenous ceftazidime or meropenem followed by an eradication phase with higher than standard doses of oral trimethoprim/sulfamethoxazole for three to six months (White et al., 1989; Cheng et al., 2009; Pitman et al., 2015). Therefore, alternative therapeutic approaches are required to treat this potentially fatal pathogen, especially the more severe forms of the disease, such as septicaemic melioidosis.

During septicaemic melioidosis, a dysregulated immune response and excessive levels of inflammation can cause damage to the host and contribute to disease, with levels of some cytokines being associated with patient mortality (Simpson et al., 2000; Wiersinga et al., 2007). Host-directed therapies act to modulate and direct host immune responses rather than directly target pathogen processes. They are promising antimicrobial candidates due to their ability to aid in pathogen clearance whilst also maintaining immune homeostasis and prevent damaging effects caused by pathogen-mediated dysregulation of the immune system (Kaufmann et al., 2018; Tripathi et al., 2021). Such therapies may prove useful in the treatment of melioidosis (Laws et al., 2019), and several therapies that attempt to re-balance or otherwise modify host responses to melioidosis have been examined and shown to have an impact on disease outcome (Cheng et al., 2007; Ceballos-Olvera et al., 2011; Asakrah et al., 2013; Charoensup et al., 2014; Laws et al., 2015; Weehuizen et al., 2016).

DZ13 is a deoxyribozyme (DNAzyme), a family of single-stranded DNA molecules with catalytic activity. DZ13 is a 34 nucleotide long single-stranded DNA (ssDNA) oligonucleotide which binds to and cleaves c-Jun mRNA, thereby modulating the activity of c-Jun (Khachigian et al., 2002). The c-Jun protein responds to diverse signals to form part of the Activating Protein-1 (AP-1) transcription factor that is involved in diverse cell responses such as proliferation, apoptosis, cell survival and tumorigenesis (Meng and Xia, 2011). Previous work has demonstrated that manipulation of this pathway can modulate host inflammation in response to infection with influenza A virus and lead to improved diseases outcomes (Xie et al., 2014; Zhang et al., 2017).

In this work, we used a murine model of *B. pseudomallei* infection to determine whether the inherent anti-inflammatory properties of DZ13 would be able to control the excessive immune response associated with the acute form of melioidosis, thus reduce morbidity and mortality.

## 2 Materials and methods

### 2.1 Oligonucleotides

The oligonucleotides used in this work are detailed in **Table 1**. These were synthesised as single stranded DNA molecules (ssDNA), purified by high-performance liquid chromatography followed by sodium salt exchange (Integrated DNA Technologies, Coralville, Iowa USA) and delivered as a dried pellet.

**Table 1:** Oligonucleotides used during this work.

Name	Sequence (5’ – 3’)	Functional c-Jun targeting domains?	Functional catalytic domain?
DZ13	CGGGAGGAA *GGCTAGCTACAACGA* GAGGCGTTG T_i_	Y	Y
ScrDZ1	GCGACGTGA *GGCTAGCTACAACGA* GTGGAGGAG T_i_	N	Y
ScrDZ2	CGGGAGGAA *GTCGTGATAGGATCG* GAGGCGTTG T_i_	Y	N
ScrDZ3	GCGACGTGA *GTCGTGATAGGATCG* GTGGAGGAG T_i_	N	N
ScrDZ4	GCGACGTGA *GTCGTGATAGGATCG* GTGGAGGAG T	N	N

The sequence of each oligonucleotide has been split across lines and are in order of 5' targeting domain, catalytic domain (italicized), 3' targeting domain. CG dinucleotides are underlined. T_i_ represents a 3′ - 3′ -linked inverted thymidine.

For use in animal studies, oligonucleotides were prepared as previously described (Xie et al., 2014) as solutions containing 1 mg/ml ssDNA (from a 10 mg/ml stock dissolved in PBS) in PBS with 5% FuGENE^®^ 6 (Promega UK) and 1mM MgCl_2_ (from a 25 mM stock; Thermo Fisher Scientific). Control mice received the same solution but with the ssDNA replaced with an equal volume of PBS. Therapies were prepared fresh on each day of treatment.

### 2.2 Animal care and welfare

Investigations involving animals were carried out according to the requirements of the UK Animal (Scientific Procedures) Act 1986. This project licence was approved following an ethical review by Dstl’s Animal Welfare and Ethical Review Body.

Studies were performed using female BALB/cAnNCrl mice (BALB/c; Charles River UK). Mice were 7 - 9 weeks of age with a mean weight at the start of procedures of 19.6 g (SD = 0.82) in study 1, 19.4 g (SD = 0.9) in study 2 and 19.8 g (SD = 0.7) in study 3. Prior to delivery (typically 3 days before shipping), mice were implanted with a sub-cutaneous Pico transponder (Uno BV, Netherlands) for identification. On arrival, mice were randomly divided into groups of five, and the groups were randomly assigned a treatment. Random numbers were generated using the standard =RAND() function in Microsoft Excel. Mice were acclimatised to their new surroundings for five days before any procedures were performed.

During this work, mice were held in an ACDP containment level 3 animal facility within a rigid-wall half-suit isolator (Howorth Air Technology, UK) supplied with an inward flow of HEPA-filtered air giving 35 to 45 air changes per hour. The room was supplied with double HEPA-filtered air giving 20 to 25 air changes per hour in the room. Mice were housed in polypropylene cages with a stainless steel mesh cover with integral water bottle holder and diet hopper which conformed to the Code of Practice for the housing of animals bred, supplied or used for scientific purposes (December 2014). Mice were under a 12-hour light/dark cycle (350 to 400 Lux during the day, 10 Lux during the night with a ramp up and ramp down period at ‘dawn’ and ‘dusk’) at 20 to 24°C and 45 to 65% relative humidity. Cages contained 8/10 and 10/14 grade corn cob (International Product Supplies, UK) as a nesting material with a range of environmental enrichment added throughout studies (e.g. irradiated aspen wood wool, cellulose dome home, hemp stem ‘Happi Mats’; International Product Supplies). There was free access to food (Labdiet certified rodent diet 5002 and Labdiet EUrodent 22% diet 5LF5; International Product Supplies) and water.

For intra-nasal challenge and treatment, mice were placed into a glass bell jar containing a small volume of isofluorane until they were lightly anaesthetised. The mice were then removed from the bell jar and 0.05 ml of solution was pipetted into the nostrils, split evenly between the two nares. Once this was fully inhaled, the mouse was placed back into its cage and monitored until consciousness was recovered and the mouse was moving freely (typically, recovery to normal behaviour would take less than a minute).

For aerosol challenges, mice were restrained within nose-only exposure tubes (CH Technologies, NJ) which were then fitted into a 20-port flow-past exposure chamber. Mice were exposed to aerosolised pathogen for 10 minutes, with a 2-minute clear-down period to remove residual pathogen from the aerosol system before mice were removed from the exposure tubes.

After challenge, mice were weighed every morning and observations made at least twice daily with any clinical signs recorded. Mice were euthanised once clinical signs had reached a point where it was deemed highly unlikely that they would be able to recover. Markers that such a point had been reached included issues with eyes, mobility, activity/alertness and/or a loss of 30% of pre-challenge weight. Mice were euthanised by cervical dislocation.

### 2.3 Animal study design

Three animal studies were conducted during this work. The studies used the same design, with the only difference being the treatments administered and the route of challenge. For studies 1 and 2, female BALB/c mice were infected with *B. pseudomallei* K96243 via the intra-nasal route on day 0. This challenge model has been described in the literature previously (Liu et al., 2002; Tan et al., 2008). In our hands using strain K96243 as the challenge agent, the LD_50_ is around 25 CFU with mortality between days 4 and 10 depending on the exact challenge dose. In these studies, a 100 CFU challenge was targeted, with actual challenges being 125 CFU and 70 CFU for studies 1 and 2, respectively. The mice received treatment via the intra-nasal route at 4 and 48 hours after infection. The dosing regimen used aligned to that previously described (Xie et al., 2014), though we chose to delay the first dose for an additional two hours (to four hours post infection) to allow more recovery time for the mice between the intra-nasal challenge and first intra-nasal treatment. At 72 hours after infection, a group of five mice from each treatment type was euthanised and organs were removed to assess bacterial burden shortly after the final treatment. The remaining ten mice from each treatment type were monitored until day 31 (study 1) or day 37 (study 2) after infection. At the end of the study, surviving mice were euthanised and organs removed to assess bacterial burden. For study 3, the intent was to follow this study design but to use an aerosol challenge. In our hands using strain K96243 as the challenge agent, the LD_50_ is less than 10 CFU with mortality between days 3 and 5 depending on the exact challenge dose (Thomas et al., 2012). However, the disease in this study was highly acute due to the high challenge and the control mice had largely succumbed by the time of the scheduled day three cull. As such, the day 3 cull was not performed and all of the animals were used for survival and monitored until day 23. This gave 15 animals per treatment group for this study, except for the mice treated with ScrDZ4 where one mouse turned in the exposure tube during challenge and was excluded from the study, leaving 14 mice in this group.

In study 1, we assessed three treatments; 1) drug vehicle, 2) DZ13, and 3) ScrDZ1. In study 2, we assessed six treatments; 1) drug vehicle, 2) DZ13, 3) ScrDZ1, 4) ScrDZ2, 5) ScrDZ3, and 6) ScrDZ4. In study 3, we assessed three treatments; 1) drug vehicle, 2) DZ13, and 3) ScrDZ4.

### 2.4 *B. pseudomallei* growth and preparation

The *B. pseudomallei* K96243 used in this study was a low passage descendant of the original stock from which genomic DNA was prepared and sequenced by Holden et al. (Holden et al., 2004). Prior to use in this study, this stock of strain K96243 was verified as being sequence type 10 using the multi-locus sequence-typing scheme of Godoy and colleagues (Godoy et al., 2003) and sequenced (Wagley et al., 2019). Virulence of this isolate has been confirmed internally, with an estimated LD_50_ at day 35 of approximately 37 CFU by the intra-nasal route and less than 10 CFU by the aerosol route (unpublished data). For counts, *B. pseudomallei* was sequentially diluted 1 ml into 9 ml PBS, spread as 0.25 ml aliquots onto LB agar, and incubated at 37°C for > 24 hours in sealed Lock & Lock boxes.

To prepare intranasal challenge material, *B. pseudomallei* K96243 was inoculated from a frozen glycerol stock into 100 ml LB-broth in a 250 ml non-baffled Erlenmeyer flask and incubated for 24 hours at 37°C with orbital shaking (180 rpm). The OD_590_ of this culture was adjusted to 0.4 (corresponding to approximately 2 x 10^8^ CFU/ml) and diluted to give a count of approximately 2 x 10^3^ CFU/ml in the challenge solution using five sequential dilutions of 1 ml into 9 ml PBS.

To prepare aerosol challenge material, *B. pseudomallei* K96243 was inoculated from a frozen glycerol stock onto the surface of a plate of LB agar and incubated for 24 hours at 37°C. The resulting growth was scraped into LB broth, adjusted to an OD of 0.4 and a 1 ml aliquot was inoculated into 1000 ml LB-broth in a 250 ml non-baffled Erlenmeyer flask and incubated for 24 hours at 37°C with orbital shaking (180 rpm). The OD_590_ of this culture was adjusted to 0.4 (corresponding to approximately 2 x 10^8^ CFU/ml) and diluted in PBS to give a count of approximately 4 x 10^6^ CFU/ml in the challenge solution.

### 2.5 *B. pseudomallei* aerosolisation and sampling

The aerosol system was controlled by an AeroMP platform (Biaera Technologies LLC (Hartings and Roy, 2004);) set to run at a negative pressure (50 to 300 Pascal) compared to the air outside of the system. The system was run at ambient temperature (19°C to 20°C) and 61 to 71% relative humidity. Vegetative *B. pseudomallei* bacteria were aerosolised from 15 ml of spray fluid by a 3-jet Collison nebulizer (CH Technologies (May, 1973)) operating at 7 L/min producing an aerosol with a particle size of 1 - 3 µm. The bacteria-laden air from the Collison was mixed with dilution air to obtain the final conditioned air at a total flow of 30 L/min.

Samples for bacterial enumeration were taken from a sampling port halfway down the exposure chamber starting 4 minutes and 30 seconds into the exposure and sampling for 1 minute. These samples were taken by impingement using all-glass impingers at 12 L/min (AGI30, Ace Glass model 7540 (May and Harper, 1957);) filled with 10 ml of PBS. Equation 1 was used to transform bacterial counts in the impinger fluid into CFU/L of air:


(1) $$Air\;conc.\;in\;CFU/L=\frac{Impinger\;conc.\;in\;CFU/ml*Liquid\;volume\;in\;ml}{Airflow\;in\;L/min*Sample\;time\;in\;min}$$

Animal challenge counts were determined by multiplying the airborne concentration of bacteria by an average mouse breathing rate (0.02 L/min determined by Guyton and colleagues (Guyton, 1947)) and then by the duration of challenge (i.e. 10 minutes). It was assumed that 40% of the inhaled dose was retained in the body as the challenge (taken from Harper and Morton (1953)).

### 2.6 Organ processing

At scheduled culls and with mice euthanised at the end of the study, the lungs, liver and spleen were aseptically removed from each mouse and weighed. These were then homogenised into 2 ml PBS through a 40 µm cell strainer using the plunger of a 5 ml plastic syringe. These organ homogenates were sequentially diluted using 0.1 ml into 0.9 ml PBS, plated as 0.25 ml aliquots onto LB agar, and incubated at 37°C for > 24 hours in sealed Lock & Lock boxes.

Following incubation, colonies on each plate where the count was 30 – 300 were counted, and these counts were used to back-calculate the concentration per ml of bacteria in the organ homogenate. The concentration of bacteria per ml was multiplied by the total volume of the organ homogenate to determine total bacterial load in that organ. The total volume of the organ homogenate was taken to be the 2 ml of PBS plus the volume contributed by the organ itself. Organ volume was calculated using the organ weight and density, where the density of the tissues of the lungs (without air), liver and spleen were assumed to be similar to the density of the human organs at 1.06 g/cm^3^ as taken from the No. 46 Report of the International Commission on Radiation Units and Measurements (ICRU, 1992). Counts below the lower limit of quantification were recorded and used in statistical analysis.

### 2.7 Assay for direct antimicrobial action

Oligonucleotides at 10 mg/ml in PBS were diluted 1 in 5 in LB-broth and twofold-diluted in LB-broth down a 96-well plate to leave 0.1 ml in each well. To each well, a 0.1 ml aliquot of *B. pseudomallei* suspension was added. To prepare this suspension, *B. pseudomallei* K96243 was inoculated from a frozen glycerol stock into 100 ml LB-broth and incubated for 24 hours at 37°C with orbital shaking (180 rpm). The OD_590_ was adjusted to 0.4 (corresponding to approximately 2 x 10^8^ CFU/ml) and diluted in LB broth to give a solution of approximately 2 x 10^6^ CFU/ml. Following addition of the *B. pseudomallei*, the 96-well plate was covered and incubated at 37°C for >24 hours in a sealed Lock & Lock box. Growth was scored visually. To control for any effects resulting from the drug vehicle, wells containing a final concentration of 1 mg/ml oligonucleotide, 5% FuGENE^®^ 6 and 1 mM MgCl_2_ were also prepared.

### 2.8 Cell culture

MH-S murine alveolar macrophage cells (ECACC, cat.#95090612) were routinely cultured to confluence in Gibco™ RPMI 1640 media with 10% foetal calf serum (FCS) and 2 mM L-glutamine in the absence of antibiotics and incubated at 37°C in a 5% CO_2_ atmosphere. Cells used in stimulation assays were kept at or below 20 passages.

HEK-Blue™ Null1 (#hkb-null1) and HEK-Blue™ mTLR9 (#hkb-mtlr9) epithelial reporter cell lines were obtained from InvivoGen (France). HEK-Blue™ Null1 cells were routinely cultured to confluence in GIBCO DMEM media with 10% FCS, 2 mM L-glutamine, 100 U/ml penicillin, 100 μg/ml streptomycin, 100 μg/ml Normocin™ (InvivoGen, #ant-nr), 200 μg/ml Zeocin™ (InvivoGen, #ant-zn) and incubated at 37°C in a 5% CO_2_ atmosphere. HEK-Blue™ mTLR9 cells were cultured under the same conditions with the addition of 20 μg/ml blasticidin (InvivoGen, #ant-bl) to media. Cells used in assays were kept at or below 20 passages.

### 2.9 Cell stimulation assays

MH-S cells were seeded into a 24-well plate at a concentration of 5x10^5^ cells/ml, with 1 ml added per well and incubated overnight to yield a final concentration of 1x10^6^ cells/ml. Cell confluence was visually assessed via microscopy prior to undertaking experiments. Wells were aspirated to remove media and 0.9 ml fresh growth media added. A 0.1 ml aliquot of 10 x test solution in growth media was then added to each well. Where test compounds were transfected, FuGENE^®^ 6 Transfection Reagent (Promega) was utilised. FuGENE^®^ 6 was mixed with oligonucleotide solution at a 3:1 ratio (μL FuGENE^®^/μg ssDNA) as previously described (Gozar et al., 2008) and incubated at room temperature for 10 minutes prior to addition to cells. Plates were incubated for 24h at which point supernatants were collected and frozen at -20°C for cytokine analysis.

### 2.10 HEK-Blue detection assays

HEK-Blue assays screening a panel of pattern recognition receptors (TLR2, TLR3, TLR4, TLR7, TLR8, TLR9, Dectin2, Mincle, NOD1, NOD2 and STING) were performed by InvivoGen as a commercial service. The assays were performed on one occasion with duplicate wells for each assay.

To quantify TLR9 stimulation in house, HEK-Blue™ Detection (Invivogen, #hb-det) culture medium was used. Cells were collected from confluent flasks of HEK-Blue™ Null1 and HEK-Blue™ mTLR9+ cells via scraping and two cell suspensions were made up in HEK-Blue™ Detection, at 2.8x10^5^ cells/mL and 2.2x10^5^ cells/mL, respectively (as per product data sheets). 20μL per well of stimulant/control compound/blank in Opti-MEM™ was added in quadruplicate (to allow duplicate wells for both cell types) to a 96-well plate. 180μl per well of each cell suspension was then added in duplicate to experimental wells. Plates were incubated at 37°C, 5% CO_2_ for 16h before being read at 620nm on a Multiskan Ascent plate reader (MTX Lab Systems).

### 2.11 Data analysis

Data was processed and visualised using Microsoft Excel 2016 and GraphPad Prism 10. Bacterial counts, ELISA data and Luminex data were assessed for normality by examining QQ plots and compared using ordinary one-way Analysis of Variance (ANOVA) with Tukey’s multiple comparisons test. For the purposes of the ANOVA, bacterial counts of zero were assigned a value at the lower limit of detection and counts below the lower limit of quantification were used directly. ELISA and Luminex results outside the limits of detection were assigned a value at the lower or upper limit of detection as needed. All bacterial counts, ELISA results and Luminex results were log10 transformed before analysis. Survival data was assessed in GraphPad Prism 10 using a Log-rank (Mantel-Cox) test.

## 3 Results

### 3.1 DZ13 and ScrDZ1 demonstrate protection against melioidosis

To assess the therapeutic efficacy of DZ13 for the treatment of melioidosis, female BALB/c mice were infected with approximately 125 CFU of *B. pseudomallei* via the intra-nasal route to establish an inhalational infection. In this model of melioidosis, several forms of disease are possible depending of the efficacy of treatment. Those mice receiving poor or no treatment develop acute disease, with clinical signs starting a few days after infection and escalating rapidly until the humane endpoint is reached. Mortality is normally within the first 10 days of infection with a peak around days 5 to 7. Those mice receiving an effective therapy do not succumb to acute disease, but can go on to develop chronic melioidosis if the therapy is only partly successful. This is characterised by prolonged but relatively moderate clinical signs, with mice sporadically succumbing to disease over a period of many weeks starting a couple of weeks after infection. In an inhalational BALB/c mouse model, complete clearance of the pathogen is difficult, so achieving sterile immunity would indicate an extremely effective therapy.

**Figure 1:**
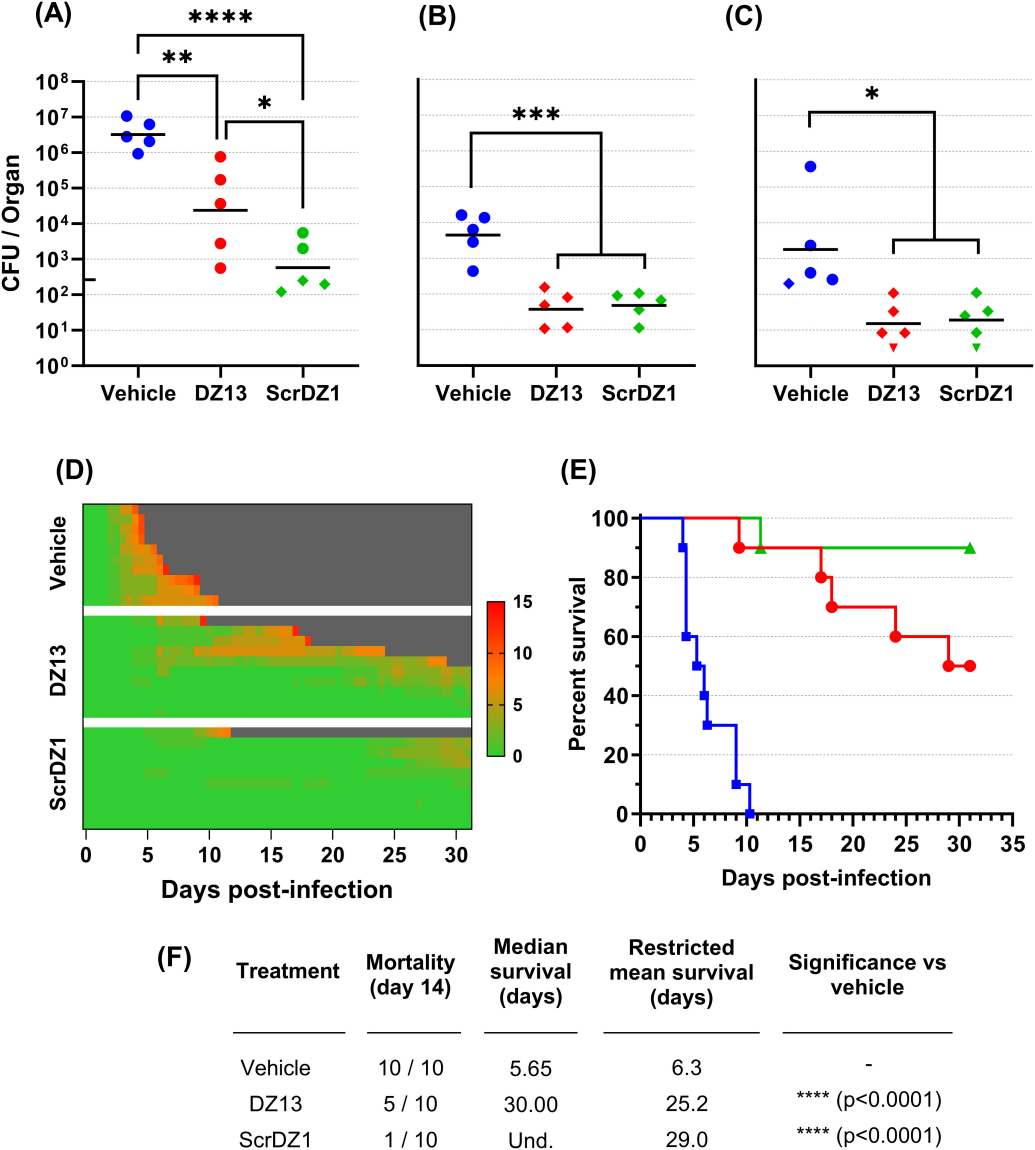
Data from animal study 1 for mice infected with *B. pseudomallei* by the intranasal route and treated with DZ13 and control substances at 4 and 48 hours after infection. Bacterial counts from the lungs **(A)**, liver **(B)** and spleen **(C)** of mice taken at day 3 post-infection. The black line indicates the geometric mean count for each treatment type. The lower limit of quantification is shown as a tick on the left-most Y-axis. Diamonds represent counts between the lower limit of quantification and lower limit of detection. Downward facing triangles represent counts below the limit of detection. Significance following an ordinary one-way ANOVA with Tukey’s multiple comparison is shown on the graph (*p<0.05, **p<0.01, ***p<0.001, ****p<0.0001). Clinical scores for infected mice are shown in panel **(D)**, coloured green to red depending on the total clinical score. Scores were recorded for coat condition, posture, mobility, activity, respiration and eye condition with each category scored 0, 1 or 2 where clinical signs were absent, mild or pronounced, respectively. Weight was scored as 0, 1, 2 or 3 where weight loss was less than 10%, 10% to 20%, 20% to 30% and greater than 30%, respectively. Grey indicates the mouse had succumbed to disease. Each line represents an individual mouse. Survival of infected mice treated with vehicle (blue, squares), DZ13 (red, circles) and ScrDZ1 (green, triangles) is shown in panel **(E)**, with survival statistics shown in the table in panel **(F)**. Und., value is undefined.

In this study, mice received therapies at 4 and 48 hours after infection. Mice receiving vehicle as a treatment developed acute melioidosis, with clinical signs of disease starting 48 hours after challenge and increasing in severity over the following days. At the day three cull (1 day after the final treatment), these mice had a high bacterial burden in the lungs with the infection having spread to distal organs such as the liver and spleen (**Figures 1A–C**). All mice in this treatment group displayed severe clinical signs and succumbed to disease by day 10 post-challenge, with a median survival of 5.65 days (**Figures 1D–F**).

Mice receiving DZ13 as the treatment did not develop acute melioidosis. Signs of disease were not observable until day four post-challenge, with clinical signs remaining at a low level in most mice for an extended period and only increasing in severity slowly (**Figure 1D**). At the day three cull, these mice had a bacterial burden in lungs, liver and spleen that was significantly lower than that present in mice treated with vehicle only (p=0.0062, p=0.0001, p=0.0101 for lungs, liver and spleen, respectively; **Figures 1A–C**). The mice in this group did not clear the infection however, and most went on to develop chronic melioidosis. By the end of the study, half of the mice had been euthanised at the humane endpoint, with a median survival for the group of 30 days (**Figures 1E, F**). There was a significant difference in survival between the mice treated with DZ13 and those treated with vehicle only (p<0.0001). All of the mice surviving to the end of the study had clinical signs and/or bacteria evident in the spleen (data not shown), and would likely have succumbed to disease eventually.

In contrast to expectations, the control oligonucleotide ScrDZ1, which lacks c-Jun targeting activity, also proved to be a highly effective treatment for melioidosis. Mice receiving ScrDZ1 did not develop acute melioidosis and clinical signs of disease remained limited for the duration of the study (**Figure 1D**). At the day three cull, these mice had a bacterial burden in lungs, liver and spleen that was significantly lower than that present in mice treated with vehicle only (p<0.0001, p=0.0002, p=0.0139 for lungs, liver and spleen, respectively; **Figures 1A–C**). Interestingly, the counts in the lungs were also lower than the counts in the lungs of mice receiving DZ13 (p=0.0336), though liver and spleen counts were not significantly different (p>0.9). Only one mouse in this group succumbed to chronic infection, and the survival in this group was significantly different to that of vehicle-treated mice (p<0.0001, **Figures 1E, F**). Six of the nine mice surviving to the end of the study had evidence of ongoing disease (clinical signs and/or bacteria in the spleen), whilst the final three mice had no clinical signs or bacteria evident in the spleen (data not shown). Interestingly, of the three treatments it was ScrDZ1 that had the best survival, with an increased survival rate compared to DZ13 (9 of 10 surviving vs 5 of 10 surviving), though this difference was not significant with the group sizes used (p=0.0687).

### 3.2 Additional ssDNA sequences also demonstrate good protection against melioidosis

The results described above were surprising for a number of reasons, but primarily because the supposedly inactive control ssDNA (ScrDZ1, lacking sequences capable of targeting c-Jun mRNA) was in fact highly protective against *B. pseudomallei*. This result would appear to be in conflict with the proposed mechanism of action by which DZ13 exerts its protective immuno-modulatory effects. To explore this finding further, we undertook a second animal study using freshly synthesised ssDNA. This study had the same overall design as previously, but included the additional treatments ScrDZ2 (functional targeting domains, non-functional catalytic domain), ScrDZ3 (non-functional targeting domains, non-functional catalytic domain) and ScrDZ4 (the same sequence as ScrDZ3 but with a standard thymidine at the 3’ end of the sequence rather than the 3’-3’-linked inverted thymidine present in the other treatments).

**Figure 2:**
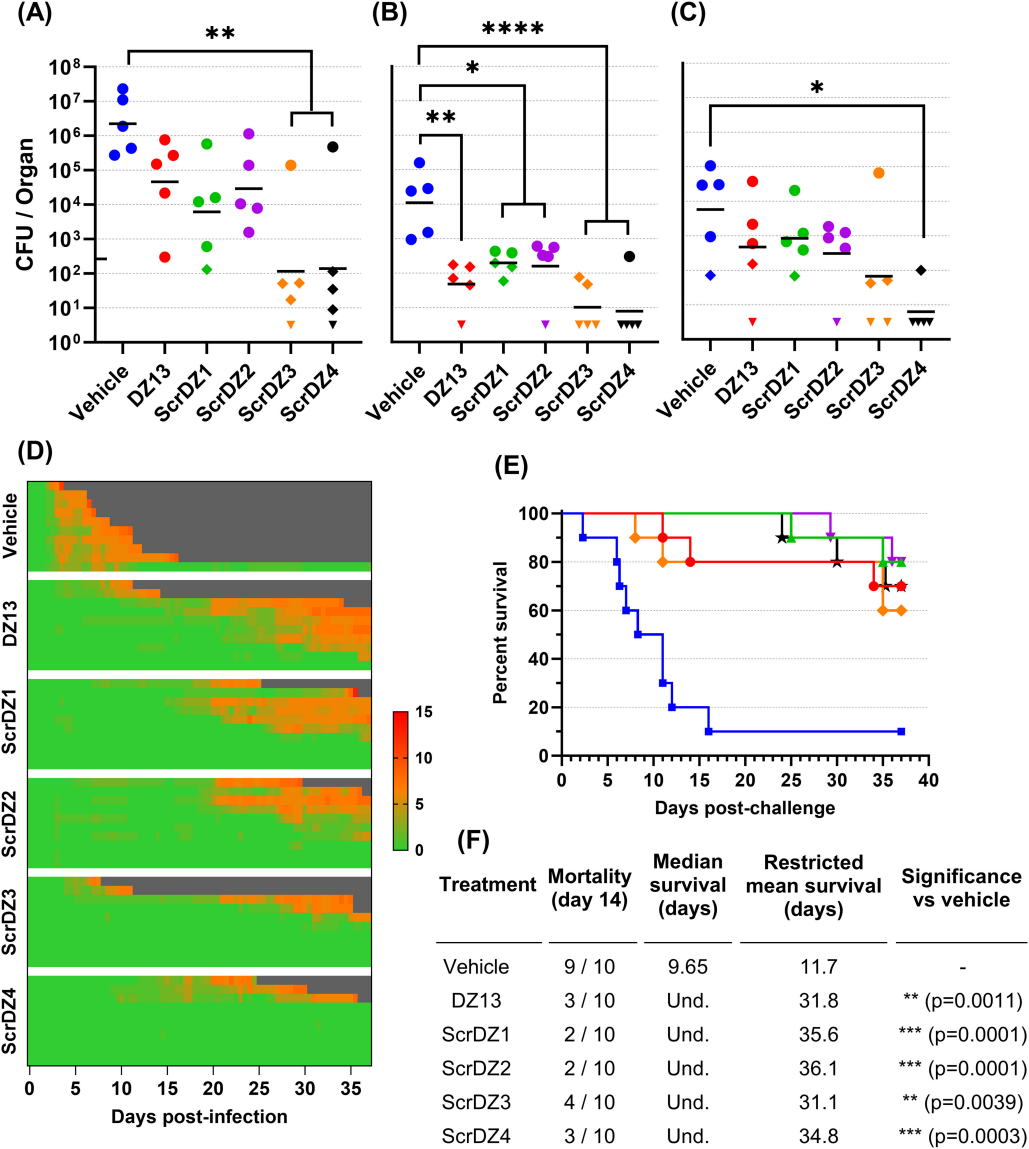
Data from animal study 2 for mice infected with *B. pseudomallei* by the intranasal route and treated with various oligonucleotides and control substances. Bacterial counts from the lungs **(A)**, liver **(B)** and spleen **(C)** of mice taken at day 3 post-infection. The black line indicates the geometric mean count for each treatment type. The lower limit of quantification is shown as a tick on the left-most Y-axis. Diamonds represent counts between the lower limit of quantification and lower limit of detection. Downward facing triangles represent counts below the limit of detection. Significance following an ordinary one-way ANOVA with Tukey’s multiple comparison is shown on the graph (*p<0.05, **p<0.01, ***p<0.001, ****p<0.0001). Clinical scores for infected mice are shown in panel **(D)**, coloured green to red depending on the total clinical score. Scores were recorded for coat condition, posture, mobility, activity, respiration and eye condition with each category scored 0, 1 or 2 where clinical signs were absent, mild or pronounced, respectively. Weight was scored as 0, 1, 2 or 3 where weight loss was less than 10%, 10% to 20%, 20% to 30% and greater than 30%, respectively. Grey indicates the mouse had succumbed to disease. Each line represents an individual mouse. Survival of infected mice treated with vehicle (blue, squares), DZ13 (red, circles), ScrDZ1 (green, triangles point up), ScrDZ2 (purple triangles point down), ScrDZ3 (orange, diamond) and ScrDZ4 (black, stars) is shown in panel **(E)**, with survival statistics shown in the table in panel **(F)**. Und., value is undefined.

The mice in this study received a challenge of 70 CFU *B. pseudomallei*, slightly lower than the first study but sufficient to cause disease. The results followed the same pattern as in the previous study. Mice receiving vehicle as the treatment at 4 and 48 hours after infection developed acute melioidosis, with clinical signs of disease starting 48 hours after challenge and increasing in severity over the following days (**Figure 2D**). These mice had high bacterial burden in organs at the day three cull (**Figures 2A–C**), and nine of the ten mice in this treatment group succumbed to disease with a median survival of 9.65 days (**Figures 2E, F**). In contrast, mice receiving any of the ssDNA treatments fared much better. These mice had clinical signs of disease starting later and for most mice remaining relatively moderate (**Figure 2D**), lower bacterial burden in organs at the day three cull (**Figures 2A–C**) and a significant improvement in survival (p=0.0011, p=0.0001, p=0.0001, p=0.0039 and p=0.0003 for pairwise comparisons between mice receiving vehicle and DZ13, ScrDZ1, ScrDZ2, ScrDZ3 and ScrDZ4, respectively, **Figures 2E, F**). Of the surviving mice, most had ongoing clinical signs and bacteria evident in the lungs and/or spleen and would likely have succumbed to disease eventually. However, there were mice having no detectable bacteria and thus had presumably cleared the infection to our ability to detect (2 of 7 mice receiving DZ13, 1 of 8 mice receiving ScrDZ1, 0 of 8 mice receiving ScrDZ2, 2 of 6 mice receiving ScrDZ3 and 5 of 7 mice receiving ScrDZ4; data not shown). None of the ssDNA treatments appeared to be greatly superior to the others in terms of survival.

### 3.3 DZ13 and ScrDZ4 are protective against an aerosol challenge

**Figure 3:**
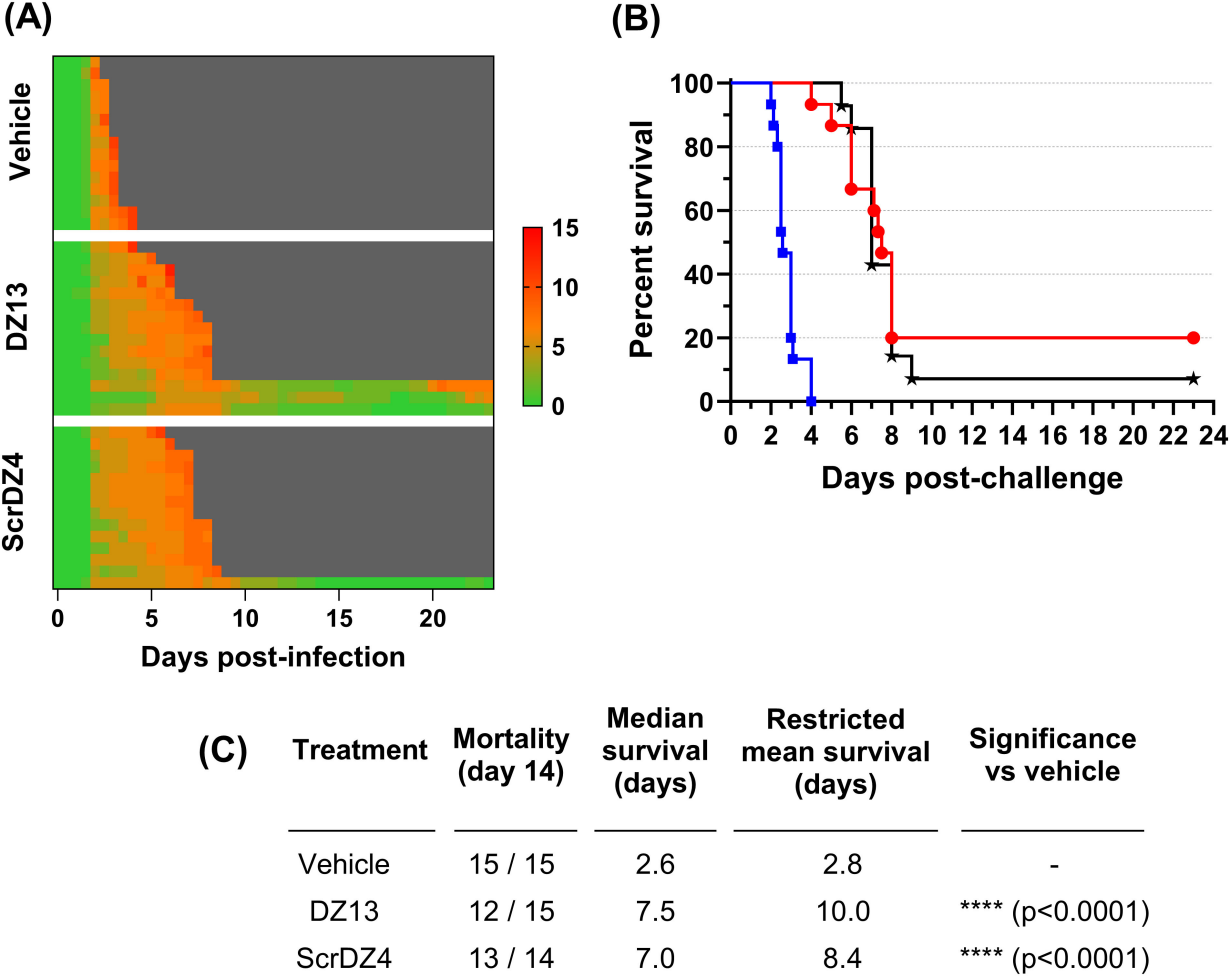
Data from animal study 3 for mice infected with *B. pseudomallei* by the aerosol route and treated with various oligonucleotides and control substances. Clinical scores for infected mice are shown in panel **(A)**, coloured green to red depending on the total clinical score. Scores were recorded for coat condition, posture, mobility, activity, respiration and eye condition with each category scored 0, 1 or 2 where clinical signs were absent, mild or pronounced, respectively. Weight was scored as 0, 1, 2 or 3 where weight loss was less than 10%, 10% to 20%, 20% to 30% and greater than 30%, respectively. Grey indicates the mouse had succumbed to disease. Each line represents an individual mouse. Survival of infected mice treated with vehicle (blue, squares), DZ13 (red, circles) and ScrDZ4 (black, stars) is shown in panel **(B)**, with survival statistics shown in the table in panel **(C)**.

To assess the protection offered by these ssDNA molecules further, we undertook a final animal study where the animals were infected with aerosolised *B. pseudomallei*. This route of challenge provides the strongest challenge for a therapeutic and is more representative of the challenge route of concern from a biodefence perspective. The mice received an average of 273 CFU as a retained dose. This was a strong challenge, and resulted in a highly acute disease. The control mice started showing clinical signs within 36 hours of challenge (**Figure 3A**) and reached the endpoint rapidly, with a median survival of 2.6 days (**Figures 3B, C**). The mice receiving DZ13 and ScrDZ4 also started showing signs of disease within 36 hours of infection, but these signs progressed more slowly and it took longer for the mice to show signs of advanced disease and reach the clinical endpoint (**Figure 3A**). Mice receiving these treatments survived for significantly longer than the control mice, with median survival of 7.5 and 7.0 days for mice receiving DZ13 and ScrDZ4, respectively (p<0.0001 for both treatments, **Figures 3B, C**). Interestingly, there were survivors in these two treatment groups, with 3 of 15 mice treated with DZ13 and 1 of 14 mice treated with ScrDZ4 surviving for three weeks after challenge (**Figure 3B**). These mice did show clinical signs during the acute phase of disease, but these signs largely disappeared after a couple of weeks. The mice were not clear of pathogen however, and several of the mice were showing signs of disease relapse at the end of the study.

### 3.4 ssDNA oligonucleotides are not directly antibacterial

With the results of the animal studies indicating that the ssDNA treatments used in this work provide protection via a mechanism other than enzymatic cleavage of c-Jun mRNA, we considered what other mechanisms might be at work. We first examined the ability of these particular ssDNA molecules to be directly antibacterial against *B. pseudomallei*. We evaluated this by exposing actively growing *B. pseudomallei* cultures to escalating concentrations of ssDNA and looking for inhibition of growth. No such growth inhibition was observed, even when *B. pseudomallei* was exposed to 1 mg/ml ssDNA (data not shown). Based on this, it is highly unlikely that the ssDNA molecules exert their effect through direct antibacterial activity.

### 3.5 ScrDZ1, ScrDZ3 and ScrDZ4 have minimal immuno-stimulatory activity

**Figure 4:**
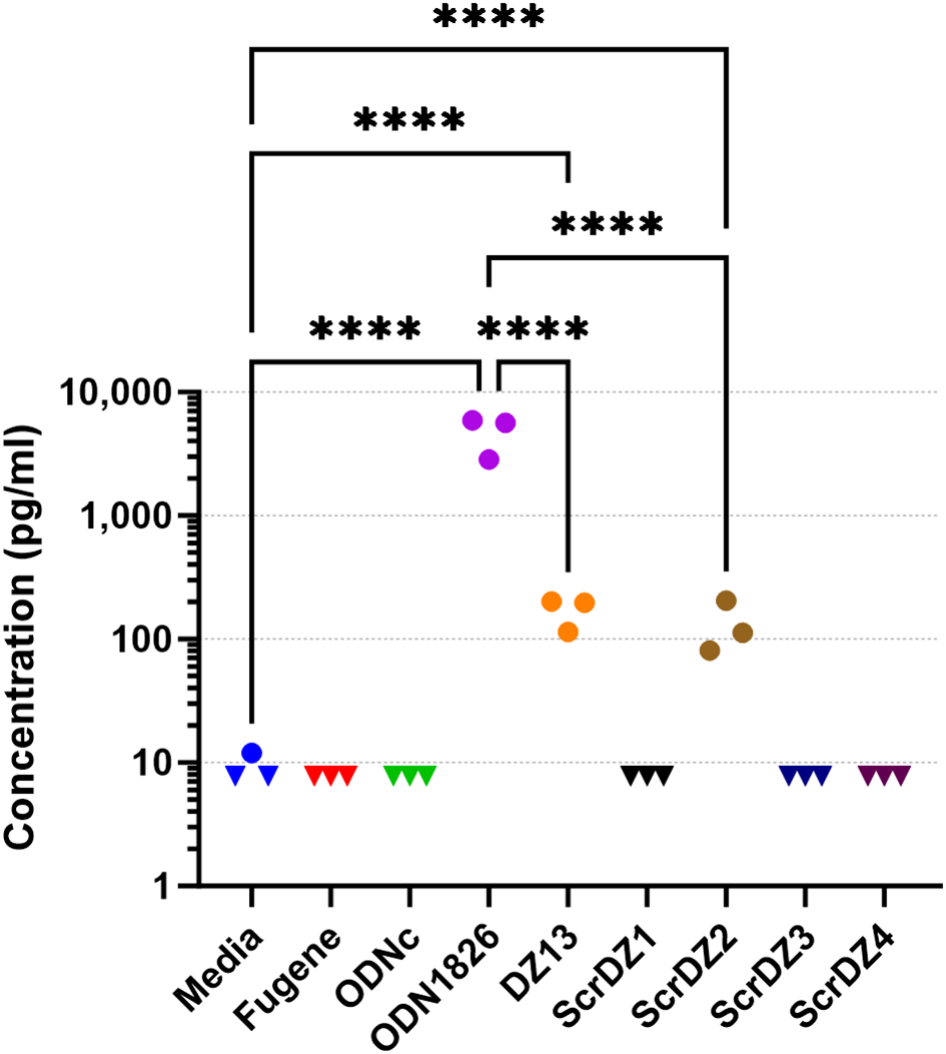
IL-6 released from MH-S cells following stimulation and quantification using an ELISA. Each assay was performed on three independent occasions, with duplicate wells for each condition in each assay. Each point on this graph is the average of the duplicate wells. Values below the lower limit of quantification are indicated by a downward facing triangle. Significance against the media only control is indicated on the graph (****p<0.0001).

With direct antibacterial activity not a factor, we next examined whether the ssDNA molecules were immuno-stimulatory and generating an environment in the host conducive to successfully combating infection. The most obvious mechanism through which this could occur was via stimulation of TLR9 by CpG-containing DNA (Hemmi et al., 2000); all of the ssDNA molecules assessed in this work contain multiple CpG dinucleotides. To assess whether these molecules had immuno-stimulatory activity, we transfected cultured immortalised mouse alveolar macrophage cells (MH-S cells) with the ssDNA molecules for 24 hours and examined the culture supernatant for secreted IL-6, a pro-inflammatory cytokine released by macrophages in response to pathogen-associated molecular patterns such as CpG-containing DNA. As expected, stimulation of the cells with a synthetic CpG-containing control molecule (ODN1826) resulted in release of high levels of IL-6 (**Figure 4**), whereas a control molecule lacking CpG dinucleotides (ODNc) resulted in no detectable release of IL-6. Of the ssDNA molecules tested, both DZ13 and ScrDZ2 resulted in significantly higher levels of IL-6 release from MH-S cells compared to unstimulated cells (p<0.0001), although these levels were significantly lower than those observed in cells stimulated with ODN1826 (p<0.0001). Interestingly, with ScrDZ1, ScrDZ3 and ScrDZ4 there was no detectable increase in levels of IL-6 (**Figure 4**).

**Figure 5:**
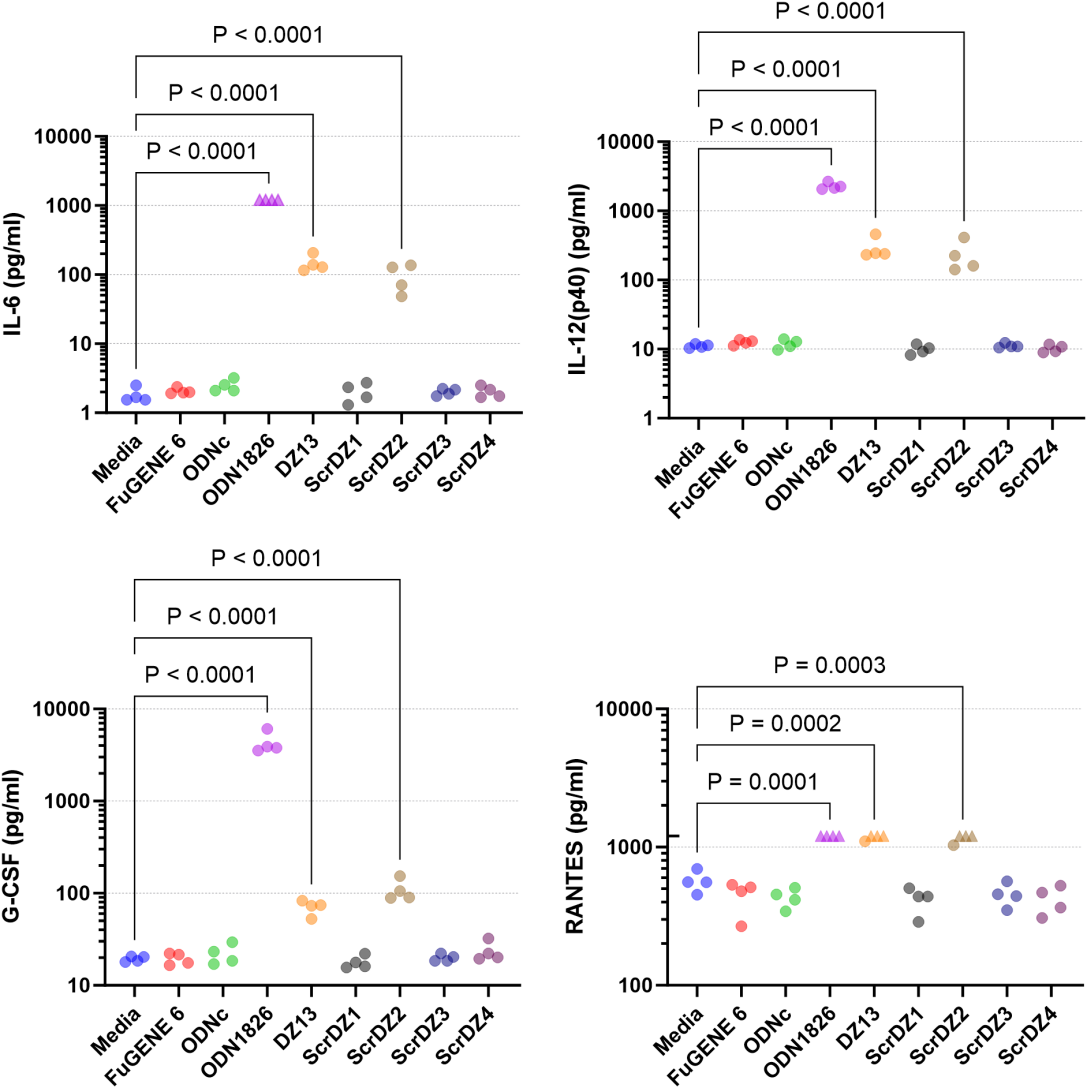
Cytokines released from MH-S cells following stimulation and quantification using Luminex technology. Selected results are shown; see **Supplementary Figure S1** for all results. Four independent cell stimulation assays were performed. Values above the upper limit of quantification are indicated by an upward facing triangle. Significance against the media only control following an ordinary one-way ANOVA with Tukey’s multiple comparison is indicated on the graph.

We considered whether there may in fact be stimulation of MH-S cells by ScrDZ1, ScrDZ3 and ScrDZ4, but that cytokines other than IL-6 were being released. To examine this, we stimulated MH-S cells with the ssDNA and performed a multiplex cytokine assay using Luminex technology. Of the twenty-three cytokines assessed, three were not quantifiable in the assay at the sample dilution used (MCP-1, MIP-1α, MIP-1β). For the remaining twenty cytokines, stimulation of cells with ODN1826 resulted in a significant increase in cytokine levels compared to untreated controls as the cells responded to the stimulant (**Figure 5**; **Supplementary Figure S1**). Significant increases in certain cytokines could also be observed in cells treated with DZ13 and ScrDZ2, though these increases were lower in magnitude than for ODN1826-treated cells. In particular, significant increases in cytokine levels compared to untreated controls were seen for IL-1α, IL-3, IL-6, IL-12(p40), IL-12(p70), G-CSF, CXCL1 and RANTES for cells treated with DZ13 or ScrDZ2 (**Figure 5**; **Supplementary Figure S1**). For cells treated with ScrDZ2 there were also significant increases in levels of IL-10, IFN-γ and GM-CSF (**Supplementary Figure S1**). For the cells treated with ScrDZ1, ScrDZ3 and ScrDZ4 however, there were no detectable increases in any of the cytokines assessed (**Figure 5**; **Supplementary Figure S1**).

**Figure 6:**
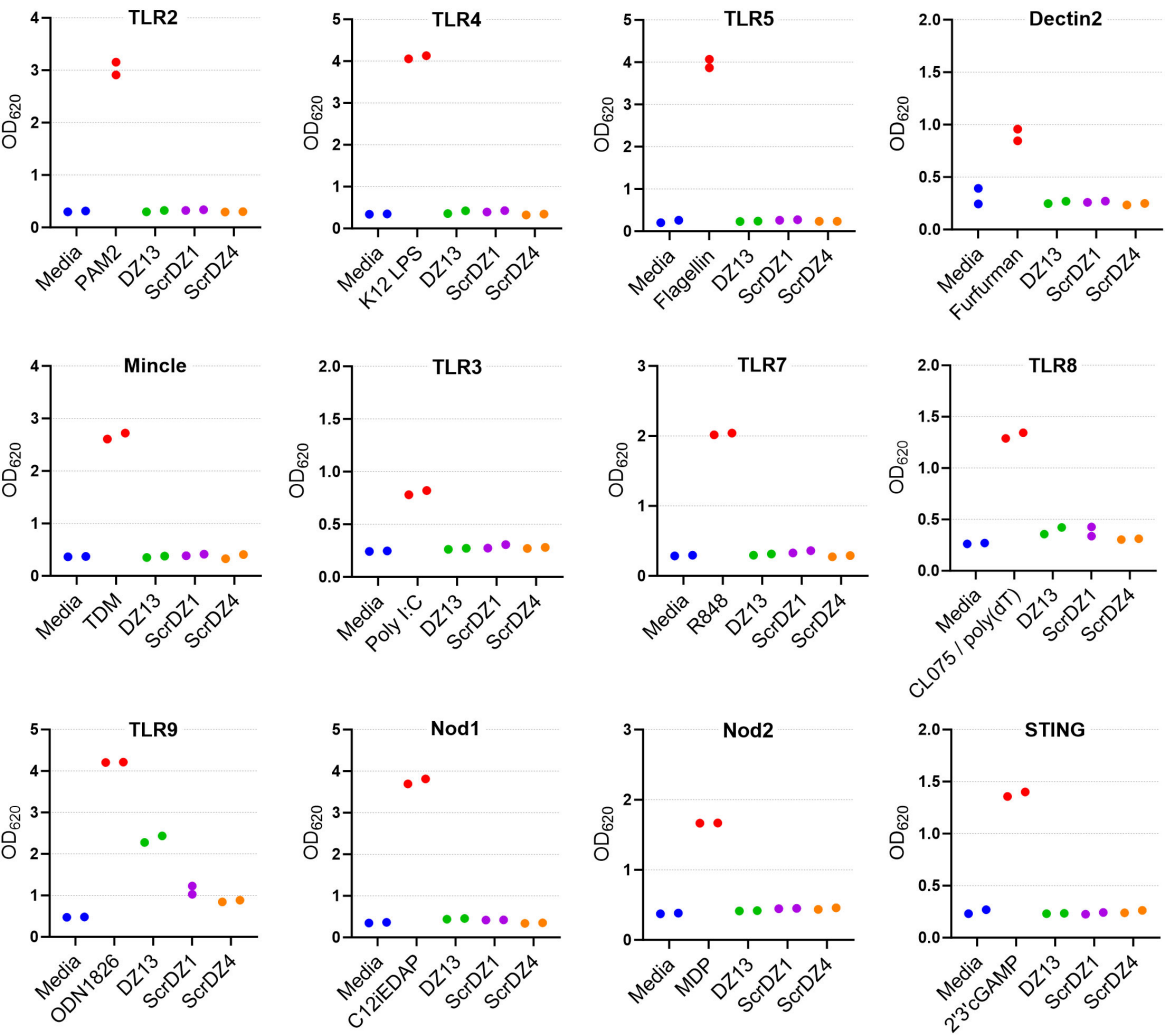
Stimulation of cells by ssDNA molecules. The pattern recognition receptor expressed by the THP-1 (STING only) or HEK-Blue cells is indicated above each graph. The positive control agonist for each cell line is indicated (red bar). Each assay was performed on one occasion with duplicate wells for each assay. The points on this graph represent the duplicate wells.

To examine this phenomenon further, we performed similar cell stimulation assays using HEK-Blue cell system. These immortalised human embryonic kidney cells stably express a reporter system whereby stimulation of the recombinant receptor expressed by the particular cell line results in production of a secreted alkaline phosphatase and generation of a blue colour that is detectable using a spectrophotometer. We examined stimulation of a panel of HEK-Blue cells expressing common pattern-recognition receptors, focussing on DZ13, ScrDZ1 and ScrDZ4. There was no stimulation evident from any of the test molecules in HEK-Blue cells expressing TLR2, TLR3, TLR4, TLR7, TLR8, Dectin2, Mincle, NOD1 and NOD2, and THP-1 cells expressing STING, whereas control agonists caused detectable levels of stimulation (**Figure 6**). For HEK-blue cells expressing TLR9, there was a large increase in optical density following stimulation by DZ13, and marginal increases in optical density following stimulation by ScrDZ1 and ScrDZ4.

**Figure 7:**
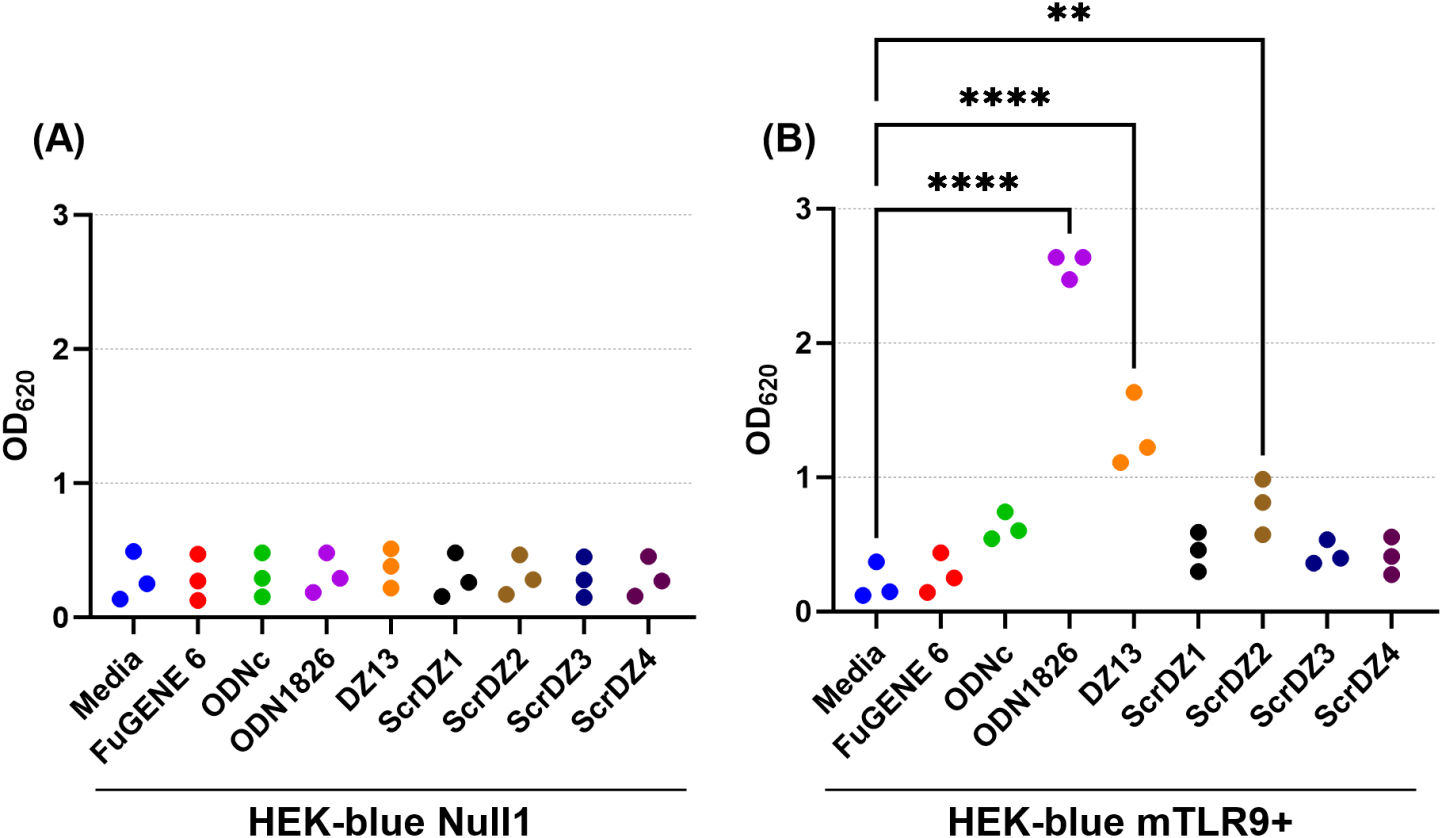
Stimulation of HEK-blue null cells **(A)** and HEK-blue TLR9+ cells **(B)** by ssDNA molecules. Each assay was performed on three independent occasions, with duplicate wells for each condition in each assay. Each point on this graph is the average of the duplicate wells in that assay. Significance against the media only control is indicated on the graph (**p<0.01, ****p<0.0001).

We wanted to understand this result further, so we performed a series of HEK-Blue assays in house focussing on HEK-blue null1 cells (i.e. not expressing a receptor) and HEK-blue mTLR9+ cells (i.e. expressing mouse TLR9) and using a wider range of test and control substances. As expected, stimulation of the HEK-blue null1 cells resulted in no detectable changes with all of the DNA molecules (**Figure 7A**), whilst stimulation of HEK-blue mTLR9+ cells with the TLR9 agonist ODN1826 resulted in strong activation of the reporter system (p<0.0001 compared to media only cells; **Figure 7B**). Stimulation of HEK-blue mTLR9+ cells with DZ13 and ScrDZ2 resulted in activation of the reporter system (p<0.0001 and p=0.0077, respectively compared to media only), though as with previous assays this activation was significantly weaker that stimulation with ODN1826 (p<0.0001). The optical density in wells of HEK-Blue mTLR9+ cells stimulated with ScrDZ1, ScrDZ3 and ScrDZ4 did appear to be marginally elevated in comparison to media only wells, though this difference was not significant (p=0.6791, p=0.7524 and p=0.8246, respectively). Indeed, the optical density of wells treated with control sequences lacking CpG motifs was also slightly elevated compared to media only, to a similar extent as with ScrDZ1, ScrDZ3 and ScrDZ4, suggesting that the marginal increase in optical density was as a result of non-specific low-level activation of TLR9 by DNA in general rather than specific activation by CpG-containing molecules. It is likely that the marginal increases in optical density from HEK-Blue mTLR9+ wells treated with ScrDZ1 and ScrDZ4 in the panel screen were also due to this phenomenon.

## 4 Discussion

The initial aim of this work was to determine whether the DNAzyme, DZ13, would provide protection against *B. pseudomallei*. Data suggests that it is a very good therapeutic in mouse models, providing excellent protection against infection, lowering bacterial burden in organs, reducing clinical signs of disease and leading to high levels of survival. This was especially pleasing since BALB/c mice are known to be highly susceptible to *B. pseudomallei* when infected via inhalational routes, indeed these models were chosen for this work in part to provide a more stringent evaluation of potential treatments than using other strains of mouse (Nelson et al., 2023). However, to our surprise the control sequence, which lacks enzymatic activity, also provided protection. This led us to develop other novel sequences, which also showed protection, including against a very strong challenge via aerosol. In terms of potential novel treatments for melioidosis then, this study provides a number of viable candidates to explore further. In particular, DZ13 might offer a ‘quick win’ in terms of translation into the clinic, having already been manufactured under GMP conditions and used in a range of animal species (Cai et al., 2012) and a small number of humans in a phase 1 trial (Cho et al., 2013). It should be noted though, that these studies used DZ13 as an intra-tumour anti-cancer agent rather than as a systemic treatment for infection, and off-target cytotoxicity has been reported (Gozar et al., 2008; Dass and Choong, 2010).

The results do pose some interesting questions about the mechanism of action for these molecules. Since all five molecules tested offered broadly equal protection against *B. pseudomallei* and are broadly similar in nature - they are all ssDNA oligonucleotides of similar length and nucleotide content - Occam’s razor would suggest the same mechanism of action was involved. Our data suggests that it is unlikely that direct antibacterial activity is involved since none of the molecules tested appear to be antibacterial even at very high concentrations. Similarly, our data suggests that the ability to degrade c-Jun mRNA is not the main mechanism of action. There is ample evidence in the literature showing that DZ13 can degrade c-Jun mRNA (Fahmy et al., 2006; Cai et al., 2012; Xie et al., 2014; Zhang et al., 2017) but the other ssDNA molecules in this study would have been incapable of degrading c-Jun mRNA because they lack functional targeting domains (ScrDZ1), a functional enzymatic domain (ScrDZ2) or both (ScrDZ3 and ScrDZ4).

CpG-mediated stimulation of the immune system would also appear to be uninvolved, or at least not the main mechanism of action based on the current understanding of CpG activity. All of the molecules tested do have multiple CpG dinucleotides, including at least one CpG that is near to the mouse consensus sequence, and we know that administration of synthetic CpG ODNs is beneficial to mice infected with *B. pseudomallei*, though usually much less effective when given post-infection rather than pre-infection (Wongratanacheewin et al., 2004; Waag et al., 2006; Rozak et al., 2010; Easton et al., 2011; Judy et al., 2012; Puangpetch et al., 2012; Puangpetch et al., 2014). However, molecules with CpG activity would be expected to be immuno-stimulatory when applied to cultured cells. Whilst DZ13 and ScrDZ2 do have some CpG-related immuno-stimulatory properties, as evidenced by release of cytokines from treated MH-S macrophage cells and stimulation of HEK-Blue mTLR9+ cells, the other three molecules seem to be inert as far as immuno-stimulation is concerned, at least in our assays using TLR9 and other pattern recognition receptors. We are currently unsure of why these sequences appear to have inactive CpGs, or rather do not behave as would be expected from traditional CpGs. It is known that CpG activity can be modulated in cis and trans by other CpG-containing sequences, including through neutralisation (Krieg et al., 1998; Zhao et al., 2000; Lenert et al., 2003), but none of the sequences in this study appear to contain known CpG neutralisation motifs. This is an active area of study.

Other “off target” effects have been reported for DZ13, including apoptosis (Gozar et al., 2008; Dass et al., 2010; Elahy and Dass, 2011) but were not directly explored in these studies as control sequences (ScrDZ1) have previously been reported to not have those properties.

Overall, our data suggests that ssDNA molecules, including DZ13, have therapeutic potential for the treatment of melioidosis. Interestingly, the molecules are not recognised by a variety of known pattern recognition receptors, except partial activation of TLR9, by two of the sequences. Clearly stimulation of TRL9 is not required for efficacy, as other sequences tested that do not stimulate that pathway, are equally effective. The data generated here using DZ13, in addition to previous findings regarding its efficacy against influenza, suggests that ssDNA molecules have the ability to be threat-agnostic therapies, which reduce the reliance on pathogen specific diagnostics and treatments. Further a broadly-acting immunomodulator has the potential to enhance survival and/or increase the therapeutic window for secondary interventions (i.e. anti-microbials) in a post-exposure setting.

## Data Availability

The raw data supporting the conclusions of this article will be made available by the authors, without undue reservation.
