# Supplementary material for: Single-stranded DNA oligonucleotides containing CpG motifs are non-stimulatory in vitro but offer protection in vivo against Burkholderia pseudomallei
Source: Front Cell Infect Microbiol. 2024 Oct 18;14:1458435. doi: 10.3389/fcimb.2024.1458435 (PMC11527787; doi:10.3389/fcimb.2024.1458435)
Supplement: Supplementary file 1 [file Table1.docx]

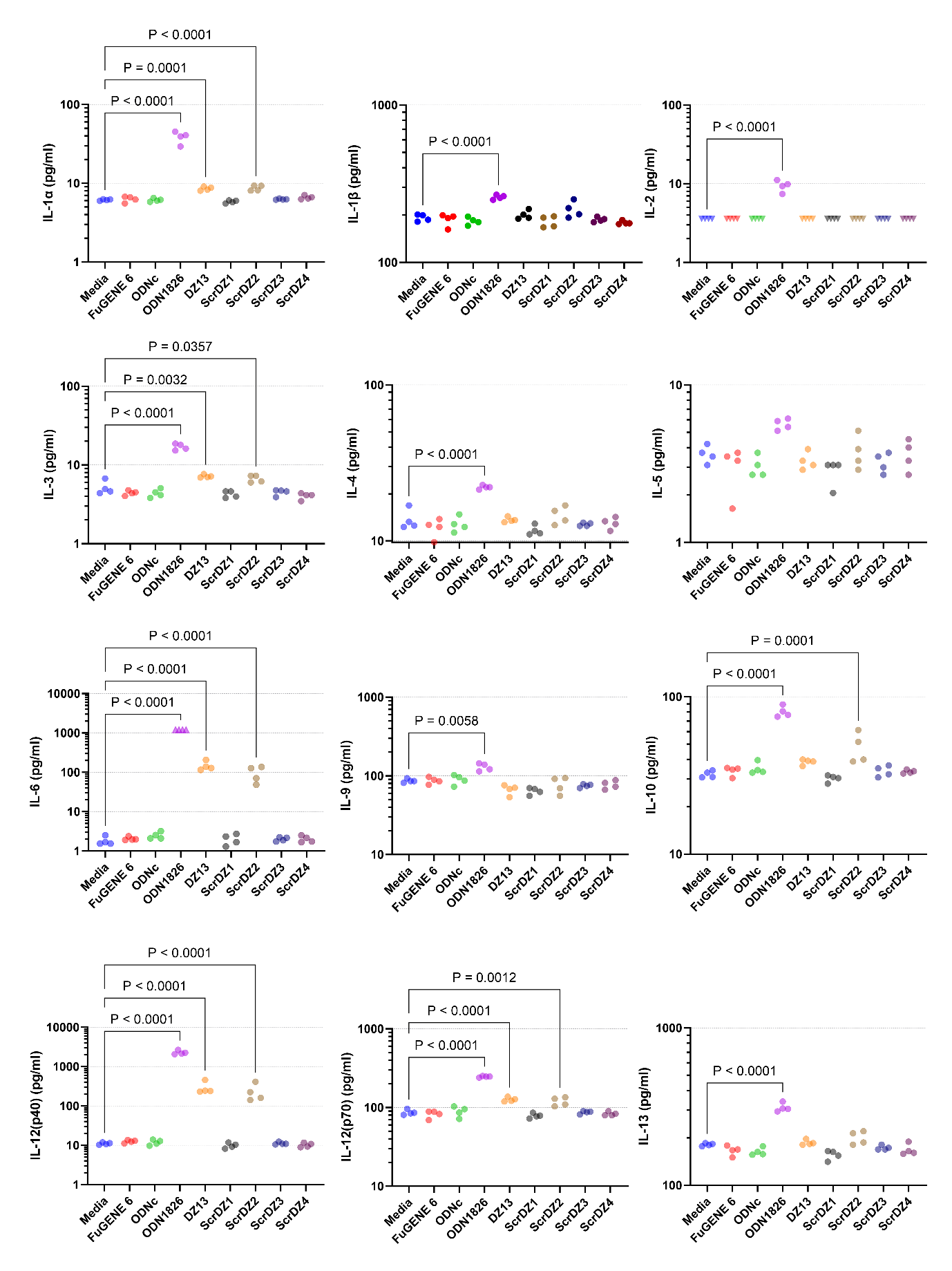


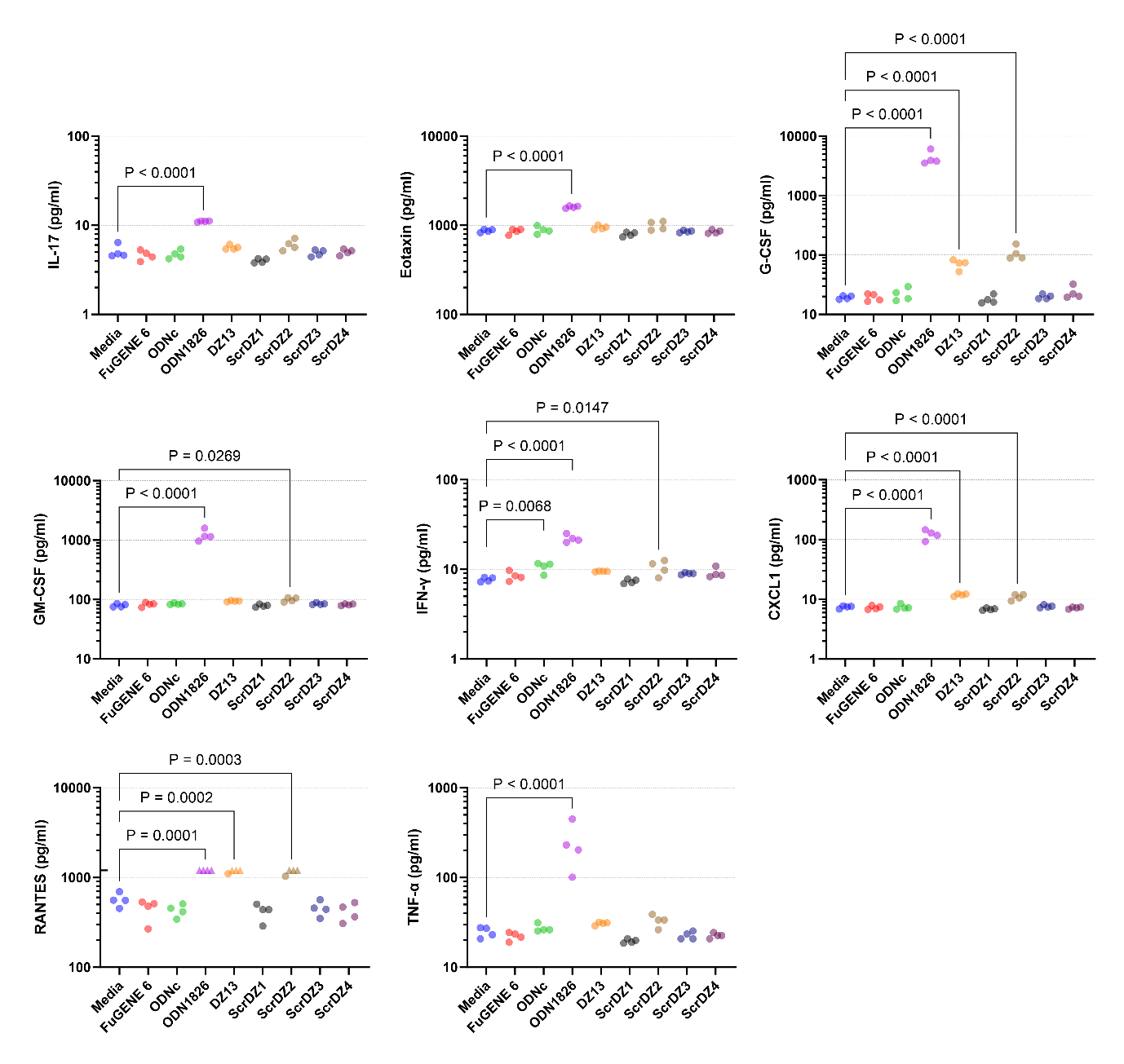


**Supplementary Figure 1.** Cytokines released from MH-S cells following stimulation and quantification using Luminex technology. Four independent cell stimulation assays were performed. Values above or below the upper or lower limit of quantification are indicated by an upward or downward facing triangle respectively. Significance against the media only control following an ordinary one-way ANOVA with Tukey’s multiple comparison is indicated on the graph.
